# The Significant Role of c-Abl Kinase in Barrier Altering Agonists-mediated Cytoskeletal Biomechanics

**DOI:** 10.1038/s41598-018-19423-w

**Published:** 2018-01-17

**Authors:** X. Wang, L. Wang, J. G. N. Garcia, S. M. Dudek, G. S. Shekhawat, V. P. Dravid

**Affiliations:** 1 Tianjin Key Laboratory of the Design and Intelligent Control of the Advanced Mechatronical System, Tianjin University of Technology, Tianjin, 300384 China; 2 National Demonstration Center for Experimental Mechanical and Electrical Engineering Education, Tianjin University of Technology, Tianjin, 300384 China; 3 Department of Materials Science and Engineering, Northwestern University, Evanston, IL 60208 USA; 4 Department of Medicine, University of Illinois, Chicago, IL 60612 USA; 5 Department of Medicine, University of Arizona, Tucson, AZ 85721 USA

## Abstract

Exploration of human pulmonary artery endothelial cell (EC) as a prototypical biomechanical system has important pathophysiologic relevance because this cell type plays a key role in the development of a wide variety of clinical conditions. The complex hierarchical organization ranging from the molecular scale up to the cellular level has an intimate and intricate relationship to the barrier function between lung tissue and blood. To understand the innate molecule-cell-tissue relationship across varied length-scales, the functional role of c-Abl kinase in the cytoskeletal nano-biomechanics of ECs in response to barrier-altering agonists was investigated using atomic force microscopy. Concurrently, the spatially specific arrangement of cytoskeleton structure and dynamic distribution of critical proteins were examined using scanning electron microscopy and immunofluorescence. Reduction in c-Abl expression by siRNA attenuates both thrombin- and sphingosine 1-phosphate (S1P)-mediated structural changes in ECs, specifically spatially-defined changes in elastic modulus and distribution of critical proteins. These results indicate that c-Abl kinase is an important determinant of cortical actin-based cytoskeletal rearrangement. Our findings directly bridge the gap between kinase activity, structural complexity, and functional connectivity across varied length-scales, and suggest that manipulation of c-Abl kinase activity may be a potential target for the treatment of pulmonary barrier disorders.

## Introduction

Numerous biological phenomena rely on conformational changes, mechanical response to external stimuli and gene-level signal transduction in both embryonic development and adult physiology. For example, the zona pellucida of mouse oocyte undergoes a “hardening” process at fertilization in order to prevent subsequent sperm from penetrating^1^. Red blood cells are known to deform extensively as they pass through tiny capillaries to deliver oxygen to remotest parts of the body^2^. Moreover, changes in local dynamic mechanotransduction of membranes or sub-cellular compartments are an important cellular response in a variety of diseases, including atherosclerosis^3^, polycystic kidney disease^4^, osteoporosis^5^, muscular dystrophy^6^, and cancer^7^. However, our understanding of how molecular and subcellular components contribute to cell stiffness, as well as how cellular mechanical properties contribute to the pathogenesis of these diseases remains limited. Thus, the discovery of localized biomechanical correlations with cellular responses is a valuable opportunity for advancing the field in a substantive way toward the development of new therapeutic approaches.

The lung endothelial cell (EC) represents a prototypical biomechanical system that participates in the dynamic regulation of the vascular size-selective barrier separating vascular contents from the interstitium and airspaces of lung. Vascular barrier disruption significantly increases permeability to fluid and proteins and occurs in many pathological conditions, including cancer, infection, sepsis, and ischemia-reperfusion injury^8^. This critical biological process requires a complex balance of mechanics. Actin filaments comprise the dynamic framework of the endothelial cytoskeleton, integrating biochemical signals and mechanical properties into a network that defines cellular structural organization and function^9^. Vascular barrier integrity is determined by the balance of actomyosin stress fiber contraction within endothelial cells, and cell-cell and cell-matrix connections that adhere these endothelial cells to each other and the underlying matrix^10–12^.

In our previous work, we investigated the relationship between cytoskeletal proteins and biomechanical properties of human pulmonary artery EC using *correlative* multiplexed microscopy, in response to thrombin, a barrier-disrupting signal, and sphingosine 1-phosphate (S1P), an endogenous barrier-enhancing agent^13^. Meanwhile, the dynamics, activity and stability of several critical kinases play important roles in actin filament rearrangement. Abl tyrosine kinases are cytoplasmic proteins that are important regulators of the actin cytoskeleton that serve to integrate multiple extracellular signals into multiple cytoskeletal responses. Therefore, they have substantial effects on force transmission, cell migration, and cellular shape^14–17^. As one of the only two member in this family known to interact directly with the cytoskeleton, the tyrosine c-Abl phosphorylates several cytoskeletal effectors that have important roles in vascular permeability and contributes to nuclear and reactive oxygen species signaling responses^18^. However, the critical role of c-Abl in regulating the actin cytoskeleton, cell-cell and cell-matrix connections, and therefore vascular permeability, is increasingly recognized but not fully characterized^19^. To unravel the innate molecule-cell-tissue length-scale relationship, in the current study we investigated the specific role of c-Abl kinase expression in endothelial cytoskeletal nano-biomechanics with atomic force microscopy (AFM) using two physiologically relevant stimuli, S1P and thrombin. Concurrently, the spatially specific arrangement of cytoskeleton structure was examined using scanning electron microscopy (SEM), and dynamic distribution of cytoskeleton related-proteins, cortactin, paxillin and VE-cadherin, were studied using immunofluorescence. The observations from both SEM and immunofluorescence serve as critical techniques to interpret the cellular mechanical properties alteration based on AFM characterization. Via endogenous c-Abl knockdown with siRNA, our study suggests that c-Abl is a key regulator of endothelial permeability altered by thrombin or S1P. This work advances the field by correlating endothelial cytoskeletal structure with biomechanical measurements in a manner that provide important mechanistic insights. This multiplexed microscopy/characterization provides novel insights into how c-Abl kinase affects cellular cytoskeleton modulation, deciphering this critical functional connection between endothelial responses and complex cytoskeletal structure for the form-function relationship.

## Methods

### Reagents and antibodies

Reagents were purchased from Sigma-Aldrich (e.g., S1P, thrombin) except as noted below. Dulbecco’s phosphate buffered saline (PBS) and trypsin were obtained from Life Technologies, while biological grade glutaraldehyde and paraformaldehyde were obtained from Electron Microscopy Science. The following reagents and antibodies were acquired for immunofluorescence: mouse monoclonal anti-cortactin (p80/85) antibody clone 4F11 (EMD Millipore), mouse anti-paxillin antibody clone 349 (BD Biosciences), mouse anti-VE-cadherin antibody (Santa Cruz), Alex Fluor 488 goat anti-mouse IgG secondary antibody (Life Technologies), HRP-conjugated β-actin antibody (Proteintech Group Inc), and Rhodamine-conjugated phalloidin (Molecular Probes). Scrambled and c-Abl specific siRNA was obtained from Santa Cruz Biotechnology, and Xfect RNA transfection reagent was obtained from Takara Clontech.

### Cell culture

Human pulmonary artery endothelial cells were purchased from Lonza and cultured in manufacturer’s recommended complete endothelial growth medium-2-microvessel (EGM-2MV, Lonza) consisting of defined growth factors and 10% fetal bovine serum (FBS, Sigma-Aldrich). Endothelial cells (EC) were maintained at 37 °C in a humidified incubator with 5% CO_2_ and utilized at passages 6–9.

### Small interference RNA (siRNA) transfection

Endogenous c-Abl expression was inhibited in ECs with specific siRNA. Briefly, c-Abl siRNA or non-targeting scrambled siRNA were transfected into ECs (passage 5–7). ECs were transfected with 0.05 μM (final concentration) c-Abl siRNA or scrambled siRNA using Xfect RNA transfection reagent per manufacturer’s protocol. After 4 hr, fresh complete growth medium was replaced and supplemented with 10% FBS and growth factors. Western blot analysis was used to assess silencing effectiveness after 48 hr. For AFM, SEM and immunofluorescence characterization, all the siRNA transfections were performed in 60 mm petri dishes. After 24 hr post-transfection, cells were detached by trypsin (0.05%) and seeded on different substrates for each measurement with desirable cell seeding concentration. ECs then grew for another 24 hr in complete growth medium prior to experimentation.

### Western blotting

After washing with -cold PBS, cells were lysed in radioimmunoprecipitation assay (RIPA) buffer [50 mM Tris-HCl, pH 7.5; 150 mM NaCl; 1% Triton X-100; 0.1% sodium dodecyl sulfate (SDS); 1% sodium deoxycholate; 0.05% NP-40; 5 mM ethylenediaminetetraacetic acid (EDTA)] with protease/phosphatase inhibitors [0.1 mM phenylmethane sulfonyl fluoride (PMSF); 1 μg/mL aprotinin; 1 μg/mL leupeptin; 10 μg/mL pepstatin; 10 mM β-glycerophosphate; 1 mM sodium fluoride; 0.1 mM sodium orthovanadate]. Cell debris was removed by microcentrifuge, and protein concentrations were quantified using Bio-Rad DC protein assay reagents. Equal amounts of protein were separated by SDS-polyacrylamide gel electrophoresis (SDS-PAGE) and transferred to nitrocellulose membranes. Membranes were incubated with primary antibodies (anti-c-Abl antibody (8E9) purchased from BD Pharmingen, San Diego, CA and HRP-conjugated β-actin antibody from Proteintech Group) in blocking solution containing 5% BSA in Tris-buffered saline-Tween 20 (TBS-T), overnight at 4 °C. Blots were washed 3 × with TBS-T, and then incubated with horseradish peroxidase-coupled secondary antibodies (Santa Cruz) in blocking solution for 60 min at room temperature. Blots were washed with TBS-T and developed using enhanced chemiluminescence (ECL) Western blotting detection reagent (Pierce, Rockford, IL). LAS-400 was used to digitize film negatives of Western blot and the band density was analyzed using ImageJ.

### AFM analysis

EC were cultured in 60 mm petri dishes (Falcon) for AFM characterization. First, cells were washed 3 × with sterile PBS to remove any debris that may stick to AFM tip during measurements. Complete medium supplemented with 2% FBS and growth factors was used to induce a basal state in the EC. AFM imaging and force measurements were performed on a 37 ° C heating stage using a BioScope Resolve AFM (Bruker), which is integrated onto an Axio Oberver.D1m (Carl Zeiss) inverted optical microscope. This system allows precise lateral positioning of the silicon nitride AFM probe over the EC of interest. A soft plastic lid is used to cover the cell-culturing petri dish with a central hole designed for AFM scanner head while performing measurements, in order to keep the cells in good condition.

The mode of PeakForce Quantitative Nanomechanical Mapping (PeakForce QNM) was used to determine morphologic change in liquid, and force-volume mode was utilized to study the mechanical properties of ECs transfected with c-Abl or scrambled siRNA after barrier alterations by S1P or thrombin. The AFM cantilever (PFQNM-LC, Bruker) spring constant was determined to be *k* = 0.06~0.09 Nm^−1^ by the thermal noise method^20^. The deflection sensitivity was calibrated by contact mode indentation on a clean glass slide (VWR International) in deionized water. Tip radius (*R*~65 nm) was obtained by scanning electron microscope (SEM, Hitachi SU8030). The loading-unloading process was performed at 6 μm.s^−1^ and accomplished within 0.25 seconds, during which the applied load, *F*, was measured as a function of the vertical actuation displacement of the piezoelectric cell, *y*, using 1024 data points. The maximum load of ~1 nN was applied at each data point to maintain cell indentations within the elastic range^21^. PeakForce QNM mode AFM image (115 μm) was rapidly acquired at a resolution of 256 lines/frame to screen for appropriate cells for subsequent experimentation. An appropriate area was selected to allow for analysis of the nucleus, cell periphery, and cytoplasmic areas to enable subcellular determination of cellular biomechanical properties. Measurements included analysis of 64 × 64 loading-unloading curves (force-volume map), with force-volume maps acquired over ~18 min. Initially a force-volume map was determined on a single transfected EC grown on the coated petri dish before stimulation. Cells were then stimulated by thrombin (1 unit/mL) followed by S1P (1 μM) during AFM measurements. Elasticity data were collected in 3 time-lapse force-volume measurements on the same cell for ~54 min (18 min × 3) for each stimulation. Thus, 7 time-lapse force-volume images were collected per experiment: 1 prior to stimulation, 3 post thrombin, and 3 post S1P. Another sample, transfected with scrambled siRNA as a control, was characterized with one time-lapse force-volume measurement before any stimulation, 3 after thrombin treatment and another 3 frames after S1P stimulation, which lasted in total ~126 min as well, in order to correspond to the time used on the c-Abl depleted sample. To assess for reproducibility, in each experiment 10 different cells were analyzed for elastic modulus determination after thrombin and S1P.

### Scanning electron microscopy (SEM) imaging

For electron microscopy, the cytoskeleton first has to be uncovered and made available to metal coating. The method of using the non-ionic detergent Triton X-100 has been adopted here to remove the cell membrane while preserving the cytoskeleton structure^22^. Transfected ECs were seeded onto a 24-well plate containing a gelatin-coated (0.25% in PBS) 12-mm glass coverslip in each well and allowed to recover in complete medium for another 24 hr posttransfection. Four coverslips were prepared in parallel from an individual passage of cells. Before any stimulation, the cells were briefly rinsed with warm sterile PBS and rendered quiescent in growth medium containing 2% FBS for 3 hr. One coverslip was with no stimulation, the other three were stimulated with either thrombin (1 unit/mL) for 18 min, S1P (1 μM) for 36 min, or thrombin (1 unit/mL) for 18 min followed by S1P (1 μM) for 36 min, in the humidified incubator. Another four coverslips with ECs transfected with scrambled siRNA as control were treated with the same set of conditions as the c-Abl-depleted ECs. After stimulation, EC were exposed to 500 μL buffer of 1% Triton X-100 and 4% PEG (MW 40,000) in stabilization buffer for 15 min, followed by 500 μL stabilization buffer containing 50 mM imidazole, 50 mM KCl, 0.5 mM MgCl_2_, and 0.1 mM EDTA (pH 7.1) for 15 min. EC were rinsed with PBS and then fixed with 500 μL of fixation solution (2.5% glutaraldehyde, 2% paraformaldehyde and 0.5% tannic acid in PBS pH 7.1) for 15 min, briefly rinsed 3× with PBS and 3× with deionized water. EC were then dehydrated through increasing ethanol concentrations of 25%, 50%, 75%, 90% and 100% for 5 min each, followed by critical point drying (Samdri-795, Tousimis). Finally, actin filament fine structure was coated with 10 nm osmium (OPC60A, Filgen), and images were captured using a SEM (Hitachi SU8030) at an accelerating voltage of 1 kV.

### Immunofluorescence confocal microscopy

ECs were seeded in 35 mm μ-Dishes with grids (ibidi GmbH) and allowed to recover in complete medium with 10% FBS for another 24 hr. Cells were washed 3× with warm sterile PBS and rendered quiescent in growth medium with 2% FBS 3 hr prior to any stimulation. Four samples were prepared simultaneously, where one coverslip acted as a control with no stimulation, the other three were stimulated with either thrombin (1 unit/mL) for 18 min, or S1P (1 μM) for 36 min, or thrombin (1 unit/mL) for 18 min followed by S1P (1 μM) for 36 min, in the humidified incubator. Another four coverslips with ECs transfected with scrambled siRNA as control were treated with the same set of conditions. After stimulation, EC were immediately fixed in 4% paraformaldehyde/PBS (pH 7.4) for 15 min and permeabilized with 0.1% Triton X-100 for 10 min. The unreacted aldehyde groups were quenched in 50 mM glycine/PBS (pH 7.4) for 15 min and the non-specific binding was blocked in 5% BSA/PBS for 30 min (pH 7.4). Incubation with primary antibody of interest (mouse anti-cortactin (1:500), mouse anti-paxillin (1:500), or mouse anti-VE-cadherin (1:250) in 5% BSA/PBS) was performed for 2 hr at room temperature. Goat anti-mouse IgG (1:500 in 5% BSA/PBS) conjugated to Alex Fluor 488 was selected as secondary antibody for 1 hr incubation at room temperature. Actin filaments were visualized by staining EC with rhodamine phalloidin for 30 min followed by nuclei staining with DAPI for 5 min with protection from ambient light. Immunofluorescent analysis was performed with an inverted laser-scanning confocal microscopy system (Zeiss Axio Observer.Z1) with a 40× oil objective lens under the exact same settings including pinhole size, detector gain, amplifier offset, scanning speed. Eight-bit images were acquired sequentially by scanning line by line with resolution of 1024 × 1024 using Zen 2009 software. Cells were representative of changes observed in three different sets of experiments. All post-acquisition image processing and quantitative analysis were performed using NIH ImageJ and Adobe Photoshop. The quantitative analysis was performed on *n* = 20 images at each condition.

## Results and Analysis

### Knockdown of c-Abl with siRNA attenuates both thrombin- and S1P-induced cell morphology alterations

**Figure 1:**
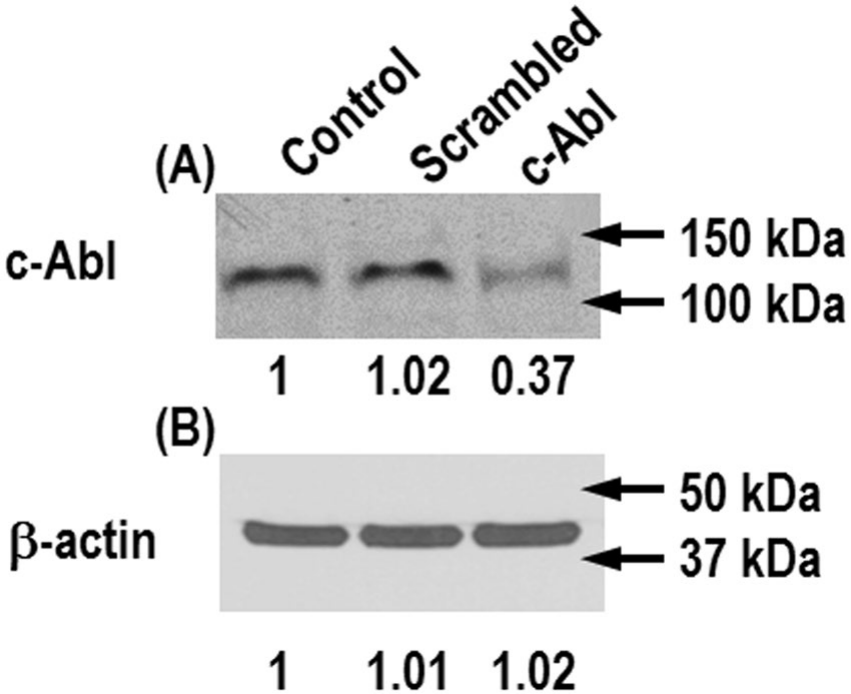
Reduction of c-Abl protein expression by siRNA transfection. ECs transfected with c-Abl siRNA were harvested 48 hr after transfection. Protein expression levels were measured by Western blot to confirm knockdown of c-Abl. Band density of the cells transfected with c-Abl siRNA was normalized relative to that of cells transfected with scrambled siRNA (**A**). A representative blot with densitometry numbers is shown. Western blot analysis of the same samples for β-actin expression demonstrates equal protein loading in each sample (**B**). Full-length blots are included in the Supplementary Information (Figure S1).

ECs were transfected with c-Abl-specific siRNA to knock down c-Abl expression to assess the specific role of this protein in EC biomechanical processes. Protein expression level was measured by Western blot to confirm knockdown of c-Abl. Band density of c-Abl depleted-EC was normalized relative to that of EC transfected with scrambled siRNA. A representative blot with densitometry numbers is shown in Fig. 1A, indicating c-Abl expression was decreased by more than 70% after 48 hr c-Abl siRNA transfection. In contrast, siRNA reduction of c-Abl did not significantly affect β-actin expression as indicated in Fig. 1B. Full-length blots are included in the Supplementary Information (Figure S1).

**Figure 2:**
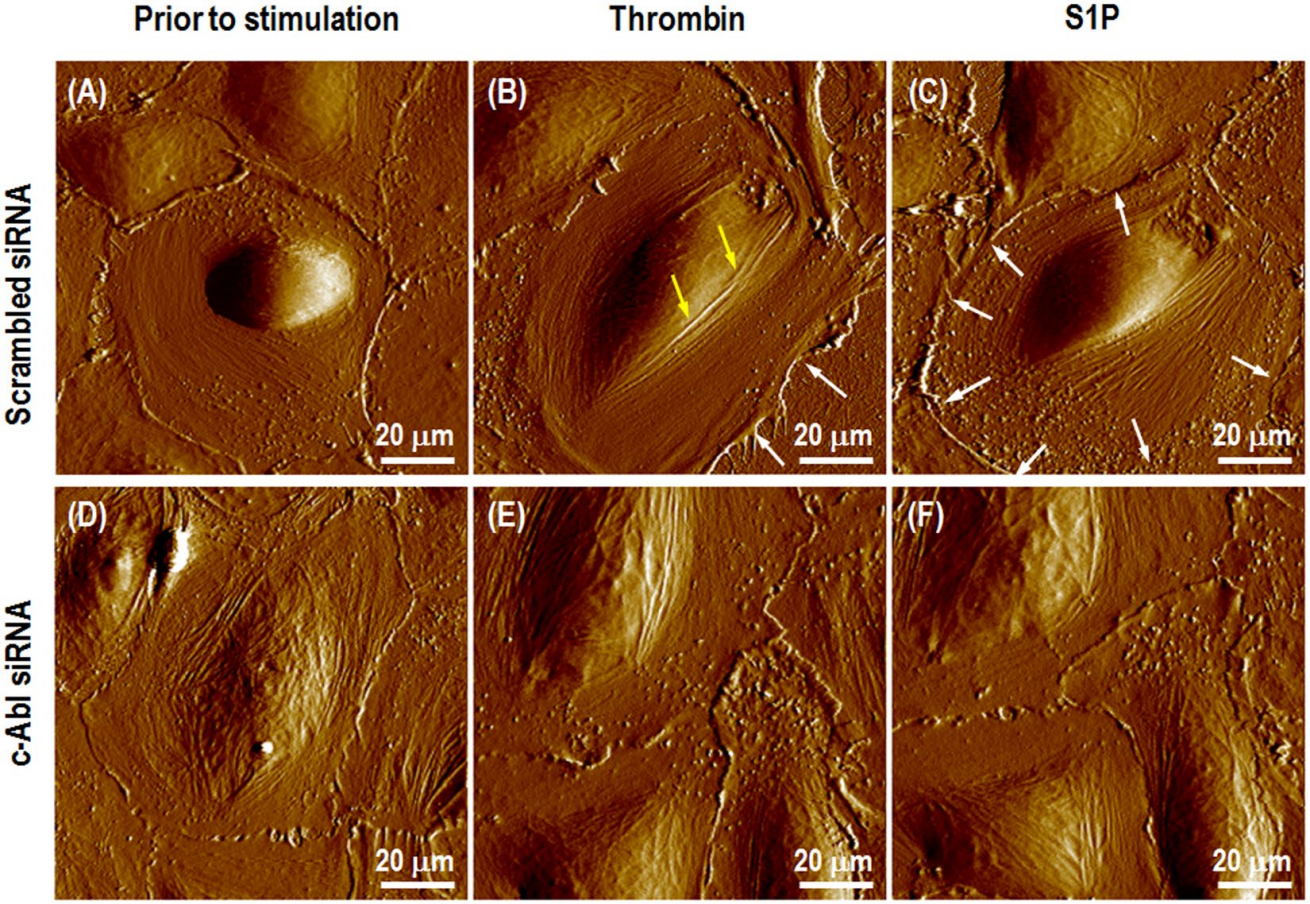
AFM scanning images of live human lung endothelial cells. Unstimulated cells transfected with scrambled, control siRNA demonstrate a polygonal shape in cobble-stone appearance without any apparent intercellular gaps (**A**); Cells were then stimulated first with thrombin (1 unit/mL, 18 min) and showed stress fibers forming over the cellular nucleus (**B**), followed by S1P stimulation (1 μM, 36 min) leading to enhanced formation of the cortical actin ring (**C**). To investigate the biological function of c-Abl kinase on cytoskeleton rearrangement, endogenous c-Abl kinase was silenced by siRNA. The monolayer of live cells transfected by c-Abl siRNA was obtained prior to any stimulation (**D**); after 18 min treated with thrombin (**E**); and then subsequent 36 min S1P challenge (**F**).

The concentration of thrombin (1 unit/mL) used is similar to the range that occurs in blood during inflammation, while 1 µM S1P substantially increases peripheral F-actin and myosin light chain phosphorylation^23,24^. Under a physiologically viable fluidic specimen stage, live ECs were analyzed with serial AFM imaging (256 lines/frame) after thrombin to induce permeability, followed by S1P to enhance barrier recovery. Figure 2 shows alterations in monolayer EC morphology after thrombin followed by S1P, using PeakForce QNM scanning mode. Lung ECs grown on plastic exhibit a polygonal morphology and normal cobble-stone appearance with mostly intact cell-cell borders as shown in Fig. 2A. After thrombin stimulation for 18 min, the cells lose contact to each other. Because the adhesive forces cannot balance the contractile forces caused by the thick actin stress fibers running over the nucleus as indicated with yellow arrows inside individual EC, large gaps are present between adjacent EC as shown in Fig. 2B. The increased cytoskeletal density in the cell center and cytoplasm and decreased adhesive forces results in the EC decreasing in size as shown with white arrows. After the addition of S1P, the cellular gaps are recovered. The peripheral actin cortex is enhanced along the perimeter of the EC, and strong interaction between adjacent cells is built through cell-cell interaction as indicated in Fig. 2C. In contrast, for the cells transfected with c-Abl siRNA, thrombin challenge fails to produce substantial stress fibers over cellular nucleus (Fig. 2E). Moreover, there is no cortical actin or other apparent enhancement in cytoskeleton density along cell periphery after S1P stimulation (Fig. 2F) in comparison to the significant rearrangements that occur after simulation in the scrambled siRNA transfected cells (Fig. 2C). These images demonstrate that c-Abl kinase plays an important role in cytoskeletal structural changes that occur in response to both barrier disruption by thrombin, and barrier enhancement by S1P.

### c-Abl siRNA inhibits thrombin- and S1P-induced changes in elastic modulus

**Figure 3:**
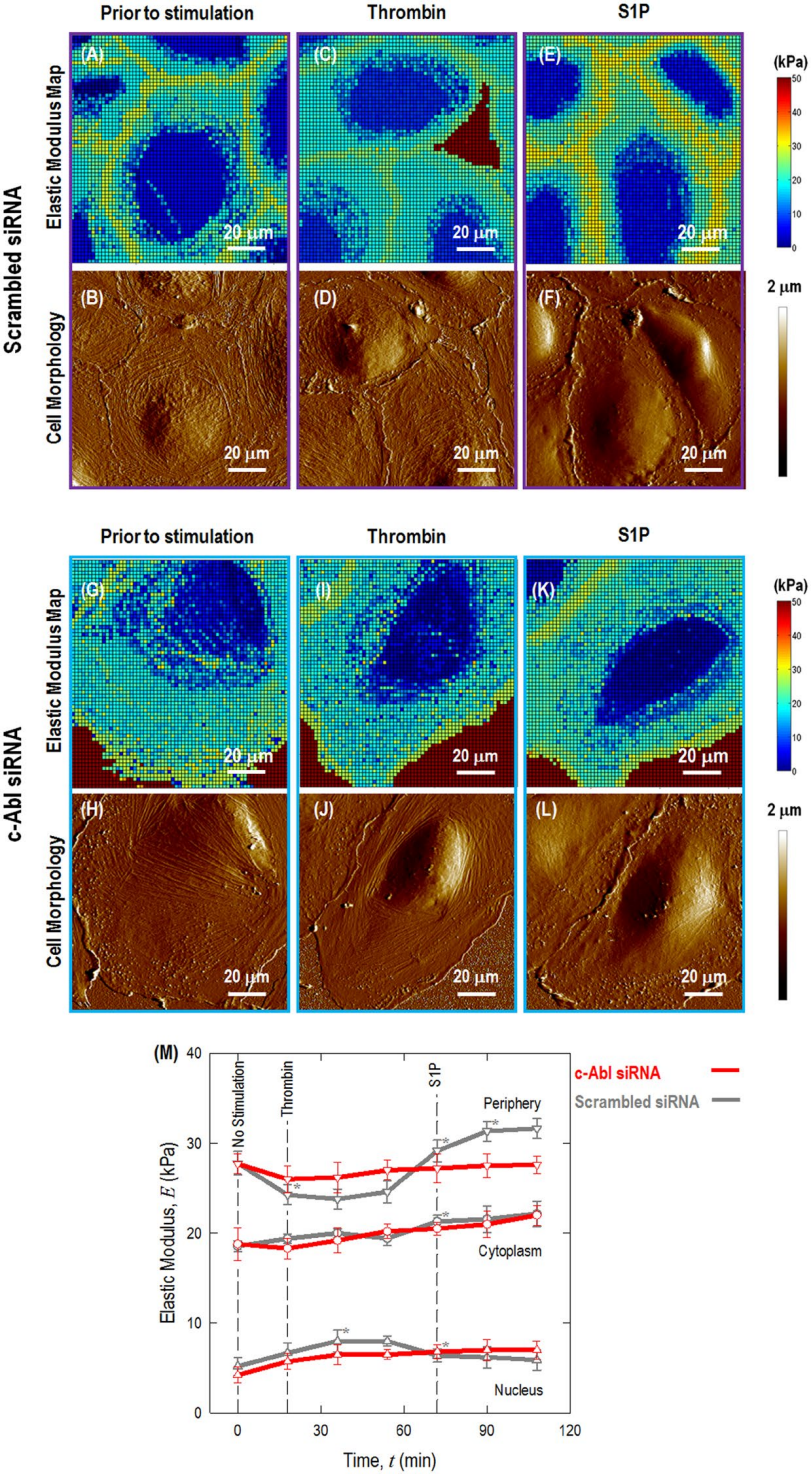
Cell morphology and time-lapse elastic modulus maps of live EC transfected with scramble and c-Abl siRNA. Representative morphology of live ECs transfected with scramble (**A**–**F**) and c-Abl siRNA (**G**–**L**) are shown prior to stimulation; after 18 min treatment with 1 unit/mL thrombin; followed by 36 min treatment with 1 μM S1P. In each experiment, 7 time-lapse force-volume images were collected: 1 for unstimulated cell (18 min), 3 after thrombin stimulation (totally 54 min) and followed by 3 after S1P stimulation (totally 54 min). The mechanical measurements were carried out by acquiring arrays of 64 × 64 loading-unloading curves in the force-volume map. Elasticity at each pixel was characterized by variable-indentation-depth curve fitting of force-displacement curve to conical Sneddon contact model using an in-house MATLAB code. (**M**) Quantification of live cell sub-cellular (periphery, nucleus, and cytoplasm) elastic modulus as a function of time in response to sequential 1 unit/mL thrombin and 1 μM S1P (as shown in red line) compared with the same cell prior to stimulation. For comparison, cells transfected with scrambled siRNA are used as control (dark grey line). *n* = 10 different cells are analyzed to generate elastic modulus time-lapse responses. Asterisks next to the points indicate statistically significant differences (^*^p < 0.05).

The distribution of elasticity in live endothelium can be determined dynamically over time with AFM force mapping. Mechanical resistance to the AFM cantilever correlates with the rigidity of the actin cytoskeleton and other structural elements, and the cellular elasticity is determined by actomyosin interaction and actin stress fiber crosslinking. Actin determines the mechanical properties of the entire cell. To assess the effects of the cytoskeleton on biomechanical properties of pulmonary EC, AFM measurements on live EC were performed before and after stimulation with thrombin and S1P. The distributions of elastic moduli (indicative of cellular resistance to the AFM probe) from the cell center to the EC periphery are analyzed in scrambled, control siRNA-transfected EC, as well as c-Abl-silenced EC. Elastic values obtained at the cell nucleus. cytoplasm, and periphery were analyzed to assess how these biomechanical properties associate with actin cytoskeletal structure observed in fluorescence microscopy and dynamic distribution of cytoskeletal-related proteins described below. Figure 3 demonstrates cellular morphology and time-lapse elastic modulus maps of live ECs transfected with c-Abl siRNA in comparison to that of cells transfected with scrambled siRNA. Figure 3 shows the cellular morphology and time-lapse elastic modulus maps of live ECs prior to any simulation, in response to 18 min treatment with thrombin and 36 min treatment with S1P.

The cellular mechanical properties are characterized using force-volume mode AFM scanning at 64 lines/frame of resolution. Elastic modulus maps were obtained by curve fitting using the Sneddon conical contact model. In addition to EC contraction induced by thrombin and cellular barrier recovery caused by S1P described above, these biomechanical assessments also provide quantitative information. Elastic modulus responses in live EC are shown in Fig. 3M. In control ECs transfected with scrambled siRNA, the elastic modulus is highest at the EC periphery, followed by the cytoplasm and nucleus. Thrombin rapidly increases stress fiber formation in the nuclear and cytoplasmic regions and EC contraction. The elastic modulus decreases significantly in the EC periphery, while the central areas increase slightly over time. This constellation of changes is associated with decreased peripheral cortical actin and increased central actin stress fibers, resulting in a contractile phenotype that produces intercellular gap formation. Maximal change in these mechanical properties occurs in the first time frame of AFM measurement after thrombin (~18 min). When S1P is added, the elastic modulus increases significantly at the EC periphery (*p < 0.05), which is associated with an increase in peripheral cortical actin that stabilizes cell-cell junctions, and counteracts thrombin to close intercellular gaps in the EC monolayer. S1P-induced EC barrier enhancement *in vitro* peaks within 30–40 min but is maintained over prolonged periods of time^23^.

However, these changes are significantly attenuated in the c-Abl-depleted ECs, with only a ~6% (not significant) decrease after thrombin challenge and then a ~7% (not significant) rise at the periphery with S1P stimulation. These data indicate that c-Abl is required for mediating the cytoskeletal alterations at the cell periphery that occur during agonist-mediated permeability changes in lung EC.

### Cytoskeleton loses representative stress fibers and cortical actin ring after c-Abl silencing

The cytoskeleton gives the cell shape and allows for adaptation to the environment under biochemical stimulation and mechanical resistance to deformation when bearing an external load^25^. The localized assembly and arrangement of the cytoskeletal architecture drive the machinery of cellular morphology changes. Visual techniques such as light and electron microscopy are critical tools for analysis of cellular structures and function in cell biology. SEM provides an intuitive method to reveal the non-labeled cytoskeleton with structural details and makes it possible to correlate cytoskeletal pattern changes in various regions with mechanical properties measurements obtained from AFM.

**Figure 4:**
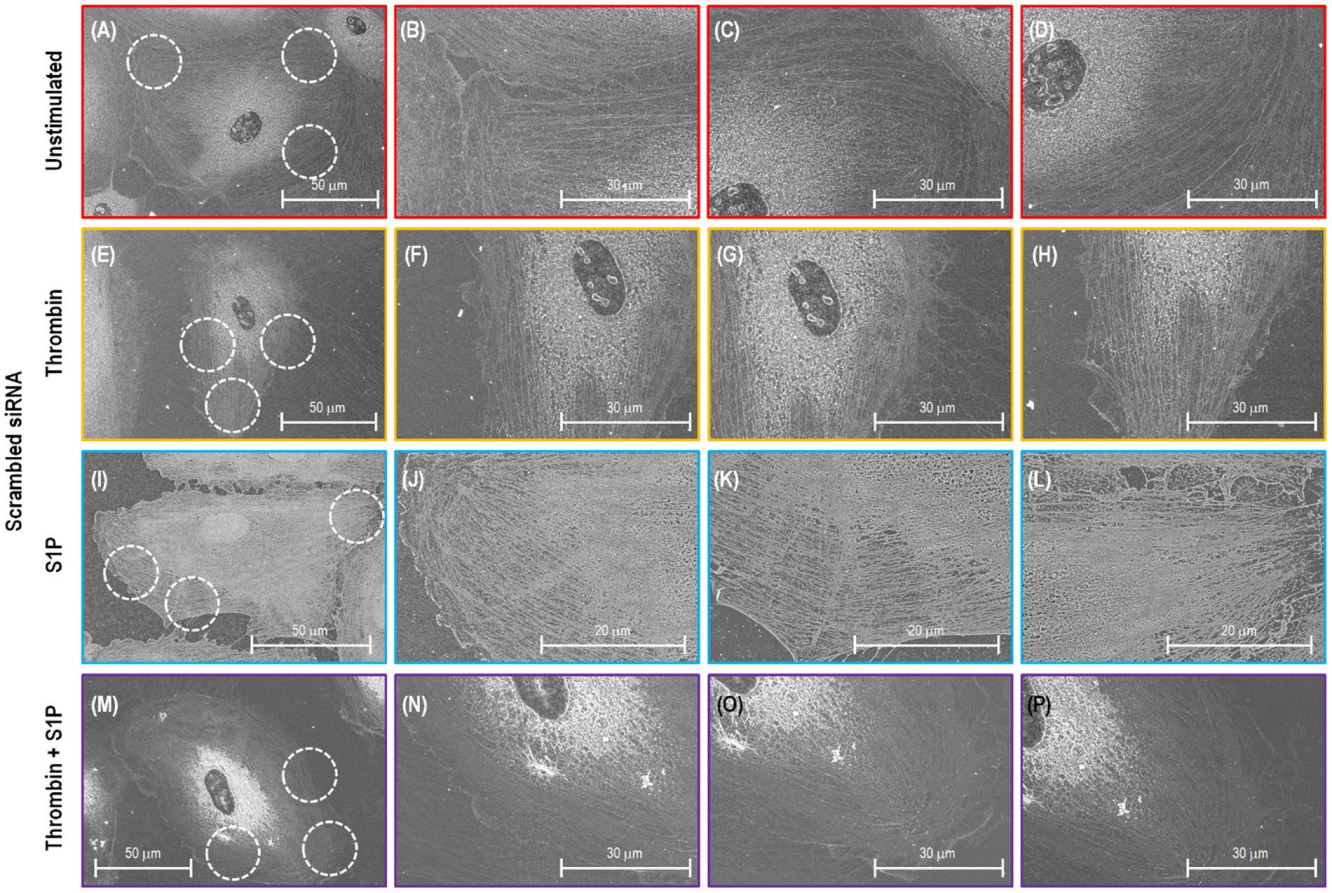
SEM images of actin filament structures of a single lung EC transfected with scrambled siRNA. Image (**A**–**D**), (**E**–**H**), (**I**–**L**) and (**M**–**P**) correspond to no stimulation, thrombin alone (1 unit/mL, 18 min), S1P alone (1 µM, 36 min) and combination of thrombin (1 unit/mL, 18 min) and followed by 36 min treated with S1P (1 µM) cells. (**B**–**D**), (**F**–**H**), (**J**–**L**) and (**N**–**P**) Are the zoomed SEM images indicating the fine structure of actin filaments in the circles with red dotted lines shown in (**A**), (**E**), (**I**) and (**M**).

Figure 4 shows SEM images of human lung EC cytoskeletal structures after extracting the cell membrane. The sample has a 10 nm osmium coating since it is a non-conductive biological cell. The SEM images of the scrambled siRNA transfected cells demonstrate that the cobblestone morphology is exhibited with few active stress fibers distributed in the cytoplasm, and a low density of fibers scatted in the periphery with random orientation (Fig. 4A–D). The thrombin-stimulated EC in Fig. 4E–H reveal substantial stress fibers. The cell morphology is contracted, and thick stress fibers are observed in the cell periphery and nuclear areas. The majority of the stress fibers are oriented in the same direction. In contrast, after stimulation with S1P (Fig. 4I–L), ECs have increased periphery actin fibers. A thick cortical band is formed at the EC periphery, which correlates to the increased peripheral elastic modulus revealed by AFM (Fig. 3). In Fig. 4M–P, the images demonstrate that ECs exhibit a high density of cytoskeleton along the cellular periphery during the S1P-induced recovery phase after initial thrombin barrier-disruption stimulation.

**Figure 5:**
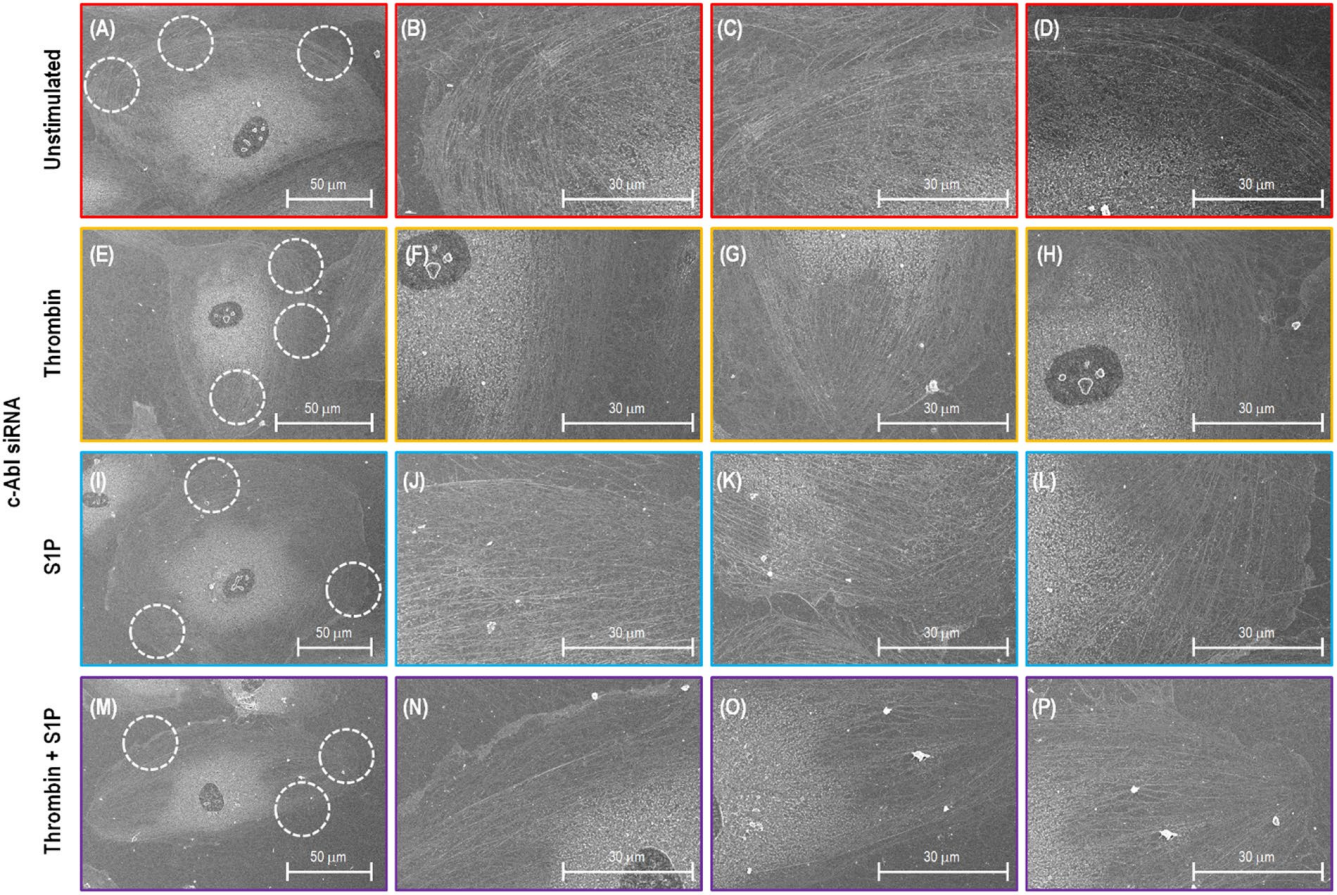
SEM images of actin filament structures of a single lung EC transfected with c-Abl siRNA. Image (**A**–**D**), (**E**–**H**), (**I**–**L**) and (**M**–**P**) correspond to no stimulation, thrombin alone (1 unit/mL, 18 min), S1P alone (1 µM, 36 min) and combination of thrombin (1 unit/mL, 18 min) and followed by 36 min treated with S1P (1 µM) cells. (**B**–**D**), (**F**–**H**), (**J**–**L**) and (**N**–**P**) are the zoomed SEM images indicating the fine structure of actin filaments in the circles with red dotted lines shown in (**A**),(**E**),(**I**) and (**M**).

Although c-Abl-silenced ECs appear to have no obvious cytoskeletal structural differences compare to control cell prior to stimulation (Fig. 5A–D), they exhibit substantially less stress fiber across cell nuclei (Fig. 5E–H), less dense actin cytoskeleton at the periphery of cells after S1P stimulation alone (Fig. 5I–L), or after the combination of thrombin and S1P (Fig. 5M–P). This correlates with significant attenuation in thrombin- and S1P-induced spatially-defined changes in elastic modulus as shown from AFM force mapping (Fig. 3). This, c-Abl siRNA substantially decreases the agent-induced cytoskeleton redistribution at the cellular periphery, demonstrating that c-Abl kinase plays a key role in the regulation of EC permeability and barrier responses. Since the extraction buffer lacks proteinase inhibitors, there are several protein debris from cell membrane shown in the SEM images.

### Knockdown of c-Abl with siRNA attenuates thrombin- and S1P-induced redistribution of paxillin, cortactin and VE-cadherin

Immunofluorescence serves as an informative approach to observe specific distribution of fluorescence-labeled proteins in three-dimensions with multi-color channels in order to reveal the dynamic assembly of the cellular frame. To provide additional insights into the biological function of c-Abl kinase on actin cytoskeletal structural rearrangement, the distribution and colocalization of the critical proteins cortactin (component of lamellipodia), paxillin (focal adhesion protein) and VE-cadherin (adherens junction protein) with the actin cytoskeleton structure were explored as follows. Actin filaments were labeled with red rhodamine-phalloidin, and blue DAPI was used as a nuclear counterstain. Meanwhile, the specific protein of interest for individual experiments (cortactin, paxillin, or VE-cadherin) was visualized by mouse antibody staining followed by a secondary antibody conjugated to Alex Fluor 488. Since detection of endogenous c-Abl in pulmonary ECs is difficult with available antibodies, it is not possible to co-label c-Abl in immunofluorescence.

**Figure 6:**
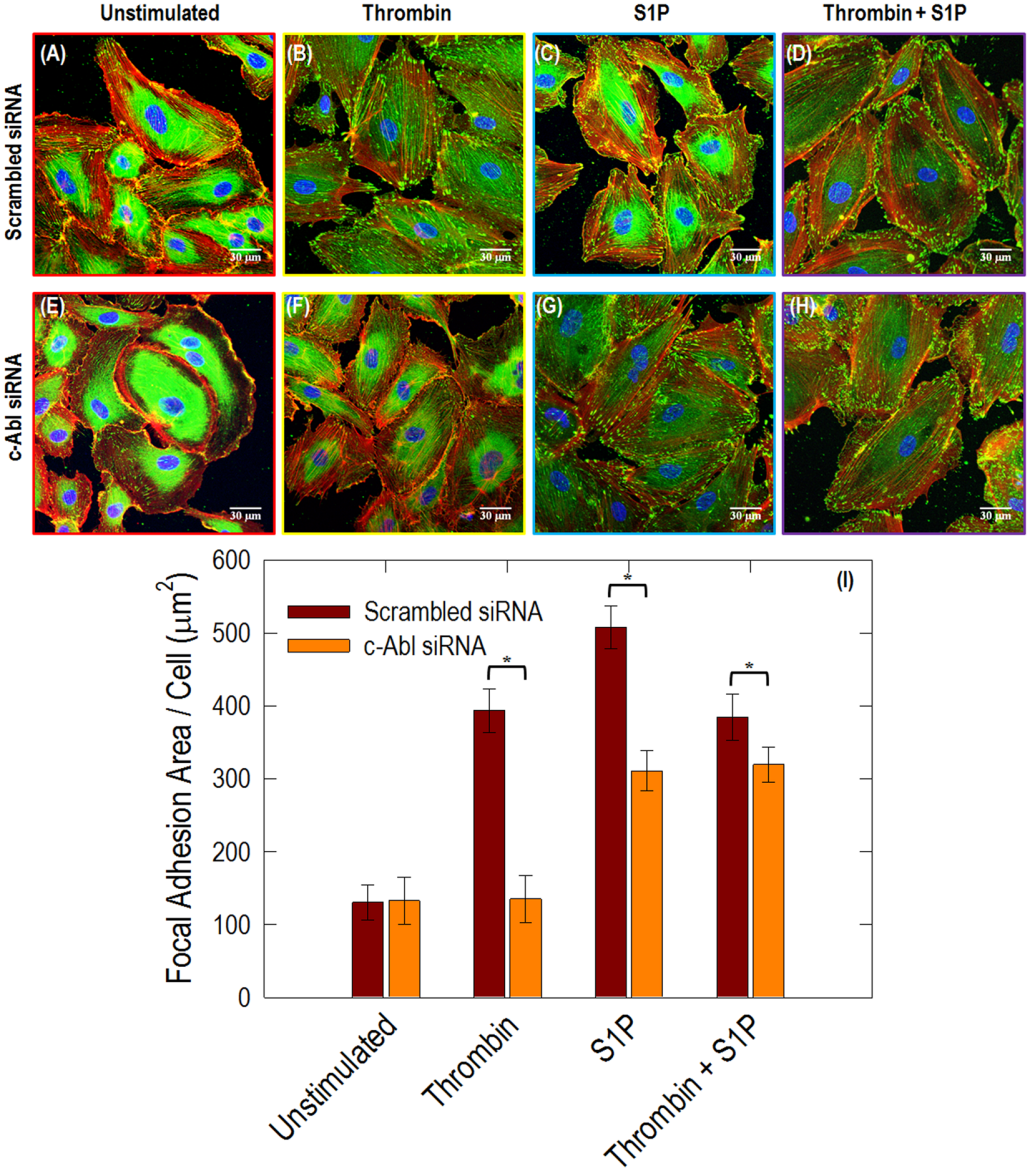
Immunofluorescence images of actin filament structures with paxillin in human lung ECs. ECs transfected with scrambled siRNA received no stimulation (**A**), thrombin alone (1 unit/mL, 18 min) (**B**), S1P alone (1 µM, 36 min) (**C**), and combination of thrombin (1 unit/mL, 18 min) followed by 36 min treated with S1P (1 µM) (**D**). ECs transfected with c-Abl siRNA received the same conditions as shown in (**E**–**H**). These are merged images between actin filament (red, stained with rhodamine-conjugated phalloidin), paxillin (green, taken with Alexa 488 fluorophore) and nuclei (blue, labelled with DAPI). The quantitative analysis was performed on *n* = 20 images at each condition using ImageJ. c-Abl depletion statistically decreases paxillin intensity in the stimulation conditions of thrombin, S1P and thrombin+S1P (*p* < 0.005).

Thrombin and S1P are known to induce dramatic cytoskeletal rearrangements. Therefore, we first investigated the effects of thrombin and S1P on paxillin rearrangement in scrambled siRNA-transfected ECs (Fig. 6A–D). Thrombin challenge induced significant accumulation of paxillin at stress fiber sites compared with unstimulated control (Fig. 6). In thrombin-treated cells, paxillin was concentrated in larger focal adhesions (394 μm^2^) compared with the smaller focal adhesions observed in untreated controls (130 μm^2^) (Fig. 6). In addition, paxillin was found primarily at the ends of stress fibers, suggesting that it was serving as an anchor for these actomyosin filaments. After S1P stimulation, paxillin staining is more intense with longer and thicker patches and more localized at the cellular periphery. Merged images of actin filament and paxillin staining further demonstrate paxillin colocalizes with the peripheral actin ring, suggesting S1P recruits paxillin along the cortical actin band. In untreated ECs transfected with c-Abl siRNA (Fig. 6E), there appear to be no obvious differences in paxillin distribution compared to control cell (Fig. 6A). However, the effects of thrombin and S1P on focal adhesion area are significantly attenuated based on quantitative analysis using ImageJ (Fig. 6I). There are fewer stress fibers after challenge with thrombin and a less prominent cortical actin band after S1P, which is consistent with the cytoskeleton structure revealed by SEM imaging (Fig. 5). In the scrambled siRNA-treated ECs before stimulation, c-Abl is primarily cytoplasmic and located along actin fibers^26^. Thrombin induces EC contraction and colocalization of c-Abl with actin stress fibers. Subsequent S1P stimulation results in multiple lamellipodia with c-Abl accumulated in these peripheral structures.

**Figure 7:**
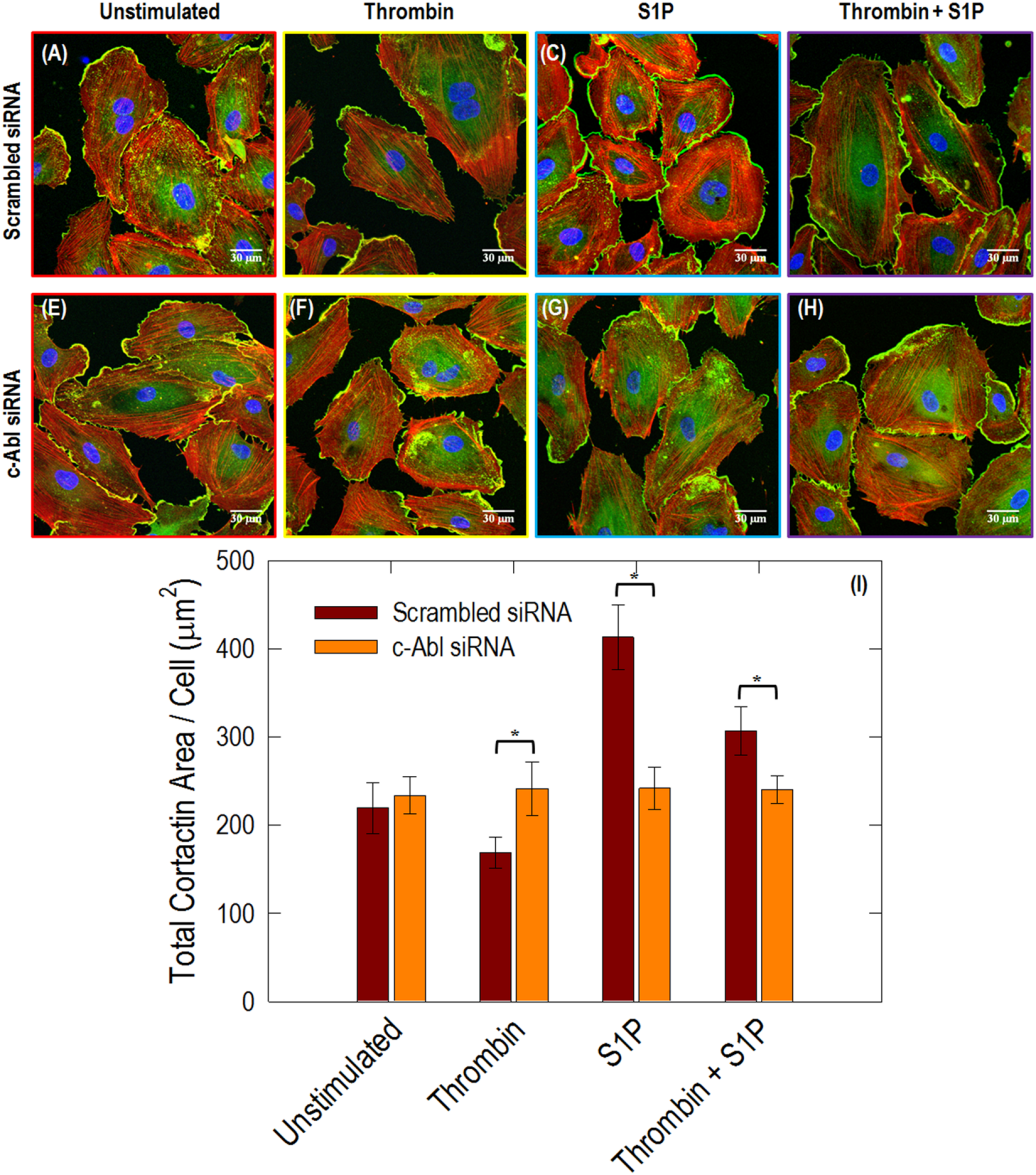
Immunofluorescence images of actin filament structures with cortactin in human lung ECs. ECs transfected with scrambled siRNA with no stimulation (**A**), thrombin alone (1 unit/mL, 18 min) (**B**), S1P alone (1 µM, 36 min) (**C**), and combination of thrombin (1 unit/mL, 18 min) followed by 36 min treated with S1P (1 µM) cells (**D**). ECs transfected with c-Abl siRNA received the same conditions as shown in (**E**–**H**). These are merged images between actin filament (red, stained with rhodamine-conjugated phalloidin), cortactin (green, taken with Alexa 488 fluorophore) and nuclei (blue, labelled with DAPI). The quantitative analysis was performed on *n* = 20 images at each condition using ImageJ. c-Abl depletion statistically decreases cortactin intensity in the stimulation conditions of thrombin, S1P and thrombin+S1P (*p* < 0.005).

Having demonstrated that c-Abl participates in paxillin redistribution after thrombin or S1P, we explored the role of c-Abl plays in cortical actin structure. Cortactin (molecular weight ~80 kDa) is an actin binding protein that can promote dynamic actin polymerization and rearrangement in the periphery^27^. Baseline confocal fluorescent microscopy with indirect immunofluorescence reveals scattered and thin peripheral and cytoplasmic cortactin staining in the unstimulated human lung EC (Fig. 7). Thrombin reduces the colocalization of cortactin with ends of actin filaments along the cellular periphery. After S1P, extensive translocation of cortactin to the cell periphery occurs as previously reported^28^. This rapid redistribution of cortactin to the EC periphery after S1P corresponds to membrane ruffling regions and colocalization with the cytoskeletal actin band, which is integral in cytoskeletal changes associated with EC barrier enhancement^28^. At baseline, c-abl siRNA has little effect on cortactin distribution at the cellular periphery since almost the same scattered pattern is observed as in the scrambled siRNA transfected cells (Fig. 7E). However, silencing of Abl substantially blocks the development of the prominent actin cortical band that is stimulated by S1P (Fig. 7G). Quantitative analysis of immunofluorescence channels (Fig. 7I) shows pooled data indicating that S1P significantly increases total cortactin area per cell. However, c-abl siRNA remarkably attenuates both thrombin- and S1P-induced cortactin rearrangements, supporting a critical role for c-Abl kinase in mediating cortactin structural formation and associated cytoskeletal rearrangements, which regulate EC barrier function.

**Figure 8:**
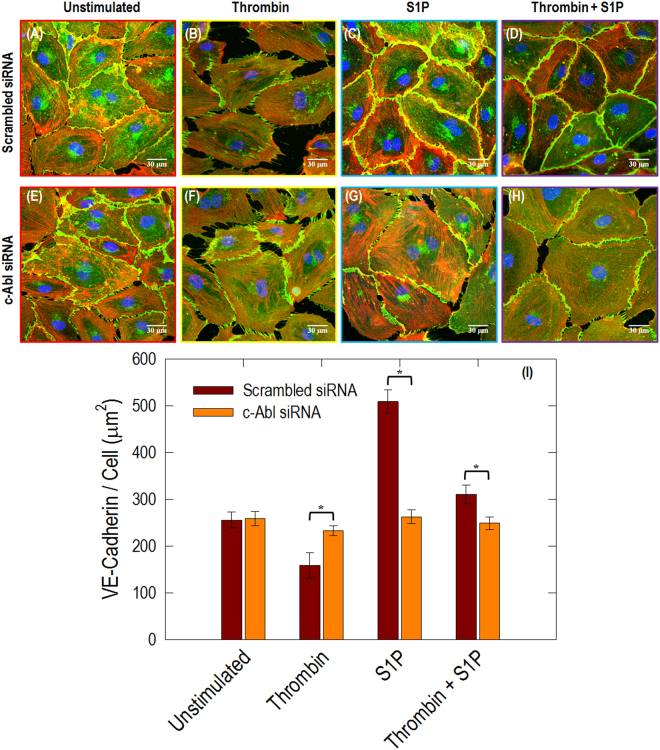
Immunofluorescence images of actin filament structures with VE-cadherin in human lung ECs. ECs transfected with scrambled siRNA with no stimulation (**A**), thrombin alone (1 unit/mL, 18 min) (**B**), S1P alone (1 µM, 36 min) (**C**), and combination of thrombin (1 unit/mL, 18 min) followed by 36 min treated with S1P (1 µM) (**D**). ECs transfected with c-Abl siRNA received the same conditions as shown in (E-H). These are merged images between actin filament (red, stained with rhodamine-conjugated phalloidin), VE-cadherin (green, taken with Alexa 488 fluorophore) and nuclei (blue, labelled with DAPI). The quantitative analysis was performed on *n* = 20 images at each condition using ImageJ. c-Abl depletion statistically decreases VE-cadherin intensity in the stimulation conditions of thrombin, S1P and thrombin+S1P (*p* < 0.005).

Intercellular junctions are a major determinant of EC permeability. Adherens junction are particularly important since their primary intercellular component, VE-cadherin, is required for maintenance of the EC barrier^29^. In the control human lung EC, the expression of VE-cadherin is concentrated in intercellular junctions in a thin pattern, showing partial continuity of adherens junctions between two cells (Fig. 8A). However, thrombin caused significant dissociation of VE-cadherin from adherens junctions in scrambled siRNA-treated EC (Fig. 8B), which is inhibited in EC by c-Abl siRNA (Fig. 8F). S1P induces accumulation of F-actin at the cell periphery and enhances continuous VE-cadherin localization at the EC periphery (Fig. 8C). However, loss of c-Abl expression alters S1P-induced enhancement of peripheral VE-cadherin, which is quantified in Fig. 8I. In combination, these results demonstrate a critical role for c-Abl in VE-cadherin distribution after thrombin and S1P stimulation.

## Discussion

The vascular endothelium is essential for mediating the profound physiologic derangements that underlie inflammatory lung injury, with increased EC permeability being a critical pathophysiologic step^24^. Alterations in important cellular mechanical properties are involved in multiple diseases^1–5^. As a result, correlating subcellular biomechanical properties correlations with EC cytoskeletal structure holds significant promise for advancing the field and increasing our knowledge in order to develop potential therapies for reversing these pathophysiologic processes.

Thrombin is a protease that is an important mediator of blood coagulation and inflammation. It increases intracellular Ca^2+^ to activate actomyosin contraction and central actin stress fiber formation in ECs^30^. In contrast, S1Pis an endogenous phospholipid that rapidly induces the peripheral localization of cortactin and myosin light chain kinase at the cell periphery to increase cell tethering forces and cell spreading to enhance the EC barrier function^31,32^. Thus, thrombin and S1P stimulate very different patterns of actin remodeling in EC.

In the present study, localized mechanical measurements from AFM (Figs 2–3), actin filament structure observation from SEM (Figs 4–5), and protein distribution from immunofluorescence (Figs 6–8) were utilized to demonstrate the central role of c-Abl kinase in regulating cytoskeletal structure and cellular biomechanical properties. Thrombin causes the formation of thick, cytoplasmic actin stress fibers and EC contraction and loss of lamellipodia. These changes result in increased elastic modulus in the central region of the cells and decreased elastic properties at the EC edges. After S1P stimulation, the actin cytoskeleton is enhanced at the cell edges, seen as membrane ruffles and cortical rings, which results in an elevated elastic modulus at the EC periphery. However, reduction in c-Abl expression by siRNA attenuates both thrombin- and S1P-mediated structural changes in human lung EC reflected by reduced stress fiber and cortical ring formation, alters the change of elastic modulus along the periphery, and decreases the peripheral relocation of cytoskeleton-related protein. This work confirms the prior report that reduction of c-Abl expression by siRNA in EC markedly attenuates S1P-mediated cortical actin formation has been studied before^26^, and it significantly advances the prior observation by investigating the effects of c-Abl kinase expression on both thrombin and S1P-mediated cytoskeleton rearrangement at multiple individual subcellular areas, such as nucleus, cytoplasm and periphery. By combining immunofluorescence with SEM, it is possible to bridge the gap between the systemic distribution of molecules/proteins of interest and corresponding cytoskeletal structural complexity in order to address the integrated feedback loop between biomechanical elements and mechanical properties from AFM characterization. Using multiplexed microscopy to correlate biophysical properties, cytoskeletal structural changes and specific protein relocation, there is evidence that EC remodeling is led by actin fiber rearrangement, which is regulated by distribution of critical cytoskeletal proteins within the cell. Overall, this work provides new insights into the important biomechanical properties of pulmonary endothelium that determine vascular barrier function. It has potential clinical applicability to pathophysiologic vascular leak syndromes such as sepsis and acute lung injury. The current study helps to address the gap between molecule dynamics, structure complexity and function connectivity. Moreover, it provides a valuable framework for understanding the form-function relationship in other biomechanical sub-systems.

## Electronic supplementary material

Supplementary information
