# Supplementary material for: The Significant Role of c-Abl Kinase in Barrier Altering Agonists-mediated Cytoskeletal Biomechanics
Source: Sci Rep. 2018 Jan 17;8:1002. doi: 10.1038/s41598-018-19423-w (PMC5772358; doi:10.1038/s41598-018-19423-w)
Supplement: Supplementary file 1 — Supplementary information [file 41598_2018_19423_MOESM1_ESM.pdf]

Supplementary for

The Significant Role of c-Abl Kinase in Barrier Altering Agonists-mediated Cytoskeletal Biomechanics

X. Wang[1, 2, 3], L. Wang[4], J. G. N. Garcia[5], S. M. Dudek[4*], G. S. Shekhawat[3*], V. P. Dravid[3*]

[1] Tianjin Key Laboratory of the Design and Intelligent Control of the Advanced Mechatronical System, Tianjin University of Technology, Tianjin, China. 300384

[2] National Demonstration Center for Experimental Mechanical and Electrical Engineering Education, Tianjin University of Technology, Tianjin, China. 300384

[3] Department of Materials Science and Engineering, Northwestern University, Evanston, IL, USA. 60208

[4] Department of Medicine, University of Illinois, Chicago, IL, USA. 60612

[5] Department of Medicine, University of Arizona, Tucson, AZ, USA. 85721

[*] Correspondence to:

sdudek@uic.edu, g-shekhawat@northwestern.edu, and v-dravid@northwestern.edu

Supplementary Materials:

Western blot

After brief washing with ice-cold PBS, cells prepared on 12-well dishes as described in the section of siRNA transfection experiments were lysed in radioimmunoprecipitation assay (RIPA) buffer [50mM Tris-HCl, pH 7.5; 150mM NaCl; 1% Triton X-100; 0.1% sodium dodecyl sulfate (SDS); 1% sodium deoxycholate; 0.05% NP-40; 5mM ethylenediaminetetraacetic acid (EDTA)] with protease/phosphatase inhibitors [0.1mM phenylmethane sulfonyl fluoride (PMSF); 1μg/mL aprotinin; 1μg/mL leupeptin; 10μg/mL pepstatin; 10mM β-glycerophosphate; 1mM sodium fluoride; 0.1mM sodium orthovanadate]. Cell debris was removed by microcentrifuge, and protein concentrations were quantified using Bio-Rad DC protein assay reagents. Equal amounts of protein were separated by SDS-polyacrylamide gel electrophoresis (SDS-PAGE) and transferred to nitrocellulose membranes. Membranes were incubated with primary antibodies (anti-c-Abl antibody (8E9) purchased from BD Pharmingen and HRP-conjugated β-actin antibody from Proteintech Group) in blocking solution containing 5% BSA in Tris-buffered saline-Tween 20 (TBS-T), overnight at 4°C. Blots were washed three times with TBS-T, and then incubated with

horseradish peroxidasecoupled secondary antibodies (Santa Cruz) in blocking solution for 60 min at room temperature. Blots were washed with TBS-T and developed using enhanced chemiluminescence (ECL) Western blotting detection reagent (Pierce). LAS-400 was used to digitize film negatives of Western blot and the band density was analyzed using ImageJ.

Full-length blots are included in the Supplementary Information as shown in Figure S1.

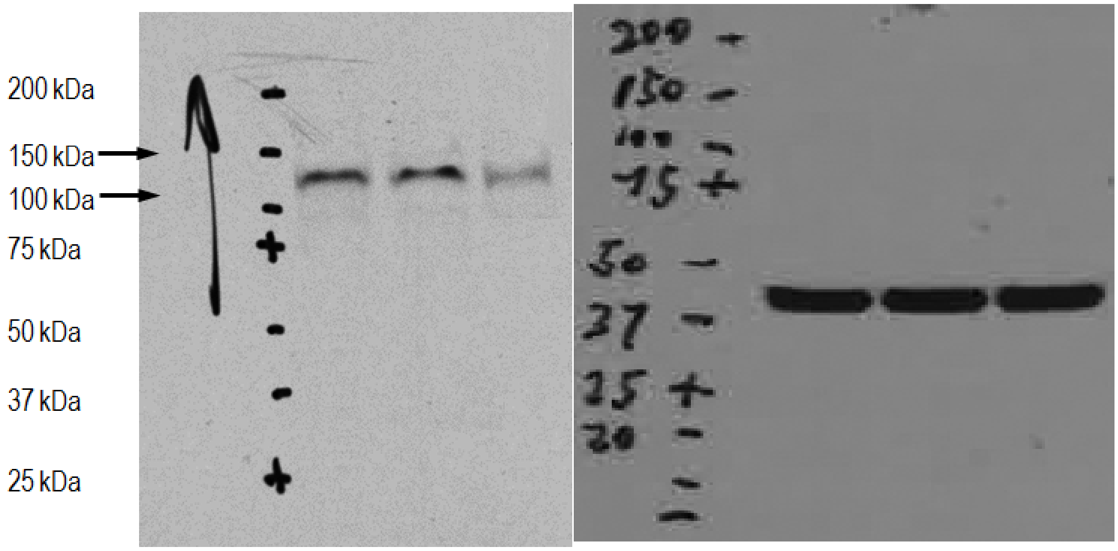

Figure S1. Full-length western blots of edited blots shown in Figure 1. (A) Western blot of c-Abl protein expression in cultured lung ECs after application of vehicle control (left lane), scramble siRNA control (middle lane), or c-Abl siRNA (right lane). (B) Samples from the same EC homogenates were analyzed by western blot for β–actin expression to demonstrate equal protein loading for each condition.

AFM imaging and mechanical measurements

ECs grown in 35 mm petri dishes were used for AFM imaging and measurements. Prior to AFM characterization, cells were generally washed three times with warmed PBS to remove any unattached staffs, in order to prevent tip contamination and improve the AFM experiments. Complete growth medium was replaced with freshly supplemented basal medium containing defined growth factors and 2% FBS. AFM imaging and force measurements were performed on a commercial system (BioScope Resolve, Bruker) with a 37°C heating stage. A soft plastic lid is used to cover the cell-culturing petri dish with a central hole designed for AFM scanner head while performing measurements, in order to keep the cells in good condition.

The mode of PeakForce Quantitative Nanomechanical Mapping (PeakForce QNM) in liquid was used to investigate the morphology alterations, and force-volume mode was utilized to study the mechanical properties of ECs transfected with c-Abl or scrambled siRNA in response to the barrier-modulating stimuli, thrombin and S1P. The spring constant of the compliant AFM cantilever (PFQNM-LC, Bruker) was measured to be k = 0.06 ~ 0.09 Nm^{-1} by thermal noise method (20). The deflection sensitivity was calibrated by repeated contact mode indentation on a clean glass slide (VWR International) in deionized water. The tip radius (R ~ 65 nm) was determined post-mortem by scanning electron microscope (SEM, Hitachi SU8030). The loading-unloading process was conducted at about 6 $\mu m.s^{-1}$ and accomplished within roughly 0.25 second, during which the applied load, F, was measured as a function of the vertical actuation displacement of the piezoelectric cell, y, with 1024 data points collected. A maximum load of ~ 1 nN was applied at each data point, in order to keep the indentations on the cells within the elastic range (21). During a typical experiment, a PeakForce QNM mode AFM image (115 μm) was rapidly acquired at a resolution of 256 lines/frame, to locate an individual cell appropriate for measurements. An appropriate area was selected containing the nucleus as well as periphery and cytoplasm in order to collect localized information of cellular mechanical properties. Measurements were carried out by acquiring arrays of 64 × 64 loading-unloading curves (force-volume map), and each force-volume map was acquired over periods of ~ 18 min. One force-volume map was first characterized on single transfected EC grown on the coated petri dish before adding any stimulation. Then cells were stimulated by sequential adding solutions of thrombin (1 unit/mL) and S1P (1 μM) during data acquisition. Elasticity measurements were collected in 3 time-lapse force-volume measurements on the same cell that lasted ~ 54 min (18 min × 3) for each stimulation. Thus for each experiment, 7 time-lapse force-volume images were collected: 1 for unstimulated cell, 3 after thrombin stimulation and 3 after S1P stimulation. Another sample, transfected with scrambled siRNA as a control, was characterized with one time-lapse force-volume measurement before any stimulation, 3 after thrombin treatment and another 3 frames after S1P stimulation, which lasted in total ~ 126 min as well, in order to correspond to the time used on the c-Abl depleted sample. To assess for reproducibility, 10 different cells were analyzed to generate the elastic modulus time-lapse in response to EC barrier-disrupting thrombin and barrier-enhancing S1P.

Scanning electron microscopy (SEM) imaging

For electron microscopy, the cytoskeleton first has to be uncovered and made available to metal coating. The method of non-ionic detergent Triton X-100 adopted here is to remove the cell membrane while preserve the cytoskeleton structure (22). Transfected ECs were seeded onto a 24-well plate containing a gelatin-coated (0.25% in PBS) 12-mm glass coverslip in each well and allowed to recover in complete medium for another 24 hr posttransfection. Four coverslips were prepared simultaneously from the same batch and passage of cells. Before any stimulation, the cells were briefly rinsed with warm sterile PBS and rendered quiescent in growth medium containing 2% FBS for 3 hr. One coverslip was with no stimulation, the other three were stimulated with either thrombin (1 unit/mL) for 18 min, S1P (1 μM) for 36 min, or thrombin (1 unit/mL) for 18 min followed by S1P (1 μM) for 36 min, in the humidified incubator. Another four coverslips with ECs transfected with scrambled siRNA as control were treated with the same set of conditions as the c-Abl-depleted ECs. After stimulation, the cells were immediately extracted with 500 μL buffer composed of 1% non-ionic detergent Triton X-100 and 4% PEG (MW 40,000) in stabilization buffer for 15 min, followed by treated with 500 μL stabilization buffer containing 50 mM imidazole, 50 mM KCl, 0.5 mM $MgCl_2$, and 0.1 mM EDTA (pH adjusted to 7.1 using concentrated HCl) for 15 min. The cells were rinsed with PBS and then fixed with 500 μL of fixation solution (2.5% glutaraldehyde, 2% paraformaldehyde and 0.5% tannic acid in PBS with final pH adjusted to 7.1) for 15 min, briefly rinsed three times with PBS and three times with deionized water. Cells were then dehydrated through a series of ascending ethanol concentrations of 25%, 50%, 75%, 90% and 100% for 5 min each, followed by critical point drying (Samdri-795, Tousimis). Finally, the actin filament fine structure was coated with 10 nm osmium (OPC60A, Filgen), and images were captured using a SEM (Hitachi SU8030) at an accelerating voltage of 1 kV.

<u>Immunofluorescence confocal microscopy</u>

ECs were seeded in 35 mm μ-Dishes with grids (ibidi GmbH) and allowed to recover in complete medium with 10% FBS for another 24 hr. Cells were washed three times with warm sterile PBS and rendered quiescent in growth medium with 2% FBS 3 hr prior to any stimulation. Four samples were prepared simultaneously, where one coverslip acted as a control with no stimulation, the other three were stimulated with either thrombin (1 unit/mL) for 18 min, or S1P

(1 μM) for 36 min, or thrombin (1 unit/mL) for 18 min followed by S1P (1 μM) for 36 min, in the humidified incubator. Another four coverslips with ECs transfected with scrambled siRNA as control were treated with the same set of conditions. After stimulation, cells were immediately fixed in 4% paraformaldehyde/PBS (pH 7.4) for 15 min and permeabilized with 0.1% Triton X-100 for 10 min. The unreacted aldehyde groups were quenched in 50 mM glycine/PBS (pH 7.4) for 15 min and the non-specific binding was blocked in 5% BSA/PBS for 30 min (pH 7.4). Incubation with primary antibody of interest (mouse anti-cortactin (1:500), mouse anti-paxillin (1:500), or mouse anti-VE-cadherin (1:250) in 5% BSA/PBS) was performed for 2 hr at room temperature. Goat anti-mouse IgG (1:500 in 5% BSA/PBS) conjugated to Alex Fluor 488 was selected as secondary antibody for 1 hr incubation at room temperature. Actin filaments were visualized by staining cells with rhodamine phalloidin for 30 min followed by nuclei staining with DAPI for 5 min with protection from ambient light. Analysis of immunofluorescent staining was performed using an inverted laser-scanning confocal microscopy system (Zeiss Axio Observer.Z1) with a 40 × oil objective lens under the exact same settings including pinhole size, detector gain, amplifier offset, scanning speed. Eight-bit images were acquired sequentially by scanning line by line with resolution of 1024 × 1024 using Zen 2009 software. Cells were representative of changes observed in three different sets of experiments. All post-acquisition image processing and quantitative analysis were performed using NIH ImageJ and Adobe Photoshop. The quantitative analysis was performed on n = 20 images at each condition.

Cellular height

On the unstimulated cells, thrombin treatment results in stress fibers assembled over the nucleus and cellular round-up, which induces an increase in cell height and EC retraction. S1P has the converse effect of flattening the cells, thus resulting in a decrease of the measured height. As shown in Table 1, the measurements represent the entire cellular heights of the scrambled siRNA transfected ECs based on measurements of 10 live cells at each condition: unstimulated 2.2 ± 0.4 µm; thrombin-treated EC 4.1 ± 0.5 µm; and S1P-treated EC 3.0 ± 0.3 µm. However, on the c-Abl transfected ECs, neither thrombin nor S1P induces significant change: unstimulated 2.3 ± 0.3 µm; thrombin-treated EC 2.6 ± 0.2 µm; and S1P-treated EC 2.5 ± 0.4 µm. Table 1 and Figure S2 show this tendency in more detail.

Table 1. Entire cellular height between scrambled and c-Abl siRNA transfected ECs

Treatment	Scrambled siRNA	c-Abl siRNA
Unstimulated	2.2 ± 0.4	2.3 ± 0.3
Thrombin (1 unit/mL, 18 min)	4.1 ± 0.5	2.6 ± 0.2
S1P (1 μM, 36 min)	3.0 ± 0.3	2.5 ± 0.4

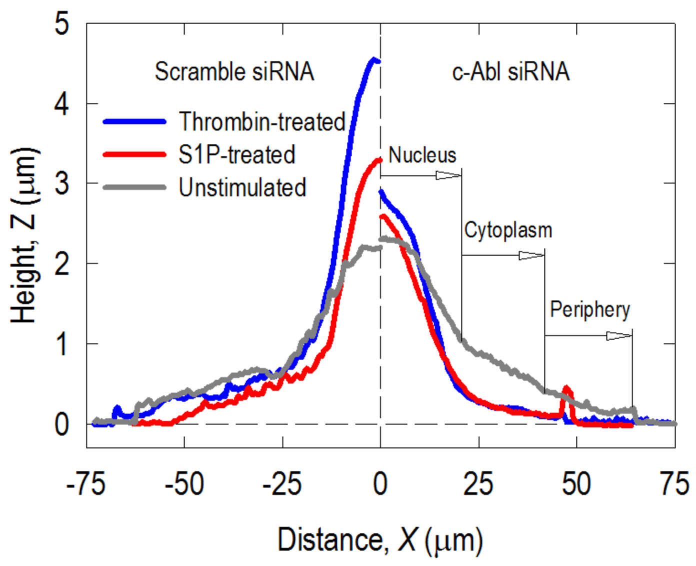

Figure S2. The tendency of cellular height at different treatment. (n = 10 cells at each condition)

<u>Data analysis</u>

In the force-volume mode, height information is also collected while performing indentation at each pixel within the scan size. The in-house developed MATLAB code read the height information from the height channel and used ~ 20% of the height at each pixel as fitting depth to fit the curve for elastic modulus. The interaction force, F, indents the cell with deformation of δ_s. According to the conical Sneddon contact mechanical model (1), the constitutive relation for a rigid conical probe with half-angle α of the indenter pressing vertically on an elastic half continuum with elastic modulus, E, and Poisson's ratio, ν = 0.50, is used to compute the cell elastic modulus. To reduce the reliability on the determination of contact point and simplify the non-linear equation, a linear version of conical Sneddon contact mechanical model is adopted (2), which is given by

$$F^{\frac{1}{2}} = \left(\frac{2}{\pi} \times \frac{E}{1-\nu^2} \times \tan\alpha\right)^{\frac{1}{2}} \times \delta_s \qquad \text{(Eqn S1)}$$

Actually, there are more than 10 cells tested per condition. During a typical experiment, a large-scale (~ 115 μm) peakforce QNM mode AFM image was rapidly acquired at a resolution of 256 lines/frame, to locate a part of monolayer ECs appropriate for measurements. Since cell may move out of the frame during ~1.5 hr treatment or cell may be dead during the scanning, thus, only 10 of cells whose results are close to the mean are used to summarize the time-lapse elastic modulus maps.

An in-house developed MATLAB (MathWorks, Inc.) code was used to obtain elasticity maps by analyzing all 4096 force curves in each force-volume mapping, where the fitting goodness values R^2 exceeded 0.85 in all curve fittings. Elastic property at each pixel was characterized by fitting of force-displacement curve to conical Sneddon contact model. Variable-indentation-depth model was developed where the curve fitting depth at each pixel was controlled within 10% of cell thickness from AFM height image in order to minimize rigid substrate effect. A line of zero force was defined from the average deflection of points on the force-displacement curve corresponding to the positions of the cantilever when it was far away from the surface.

Quantitative analysis on immunofluorescence images

To obtain quantitative analysis on how c-Abl kinase silence with siRNA affects the formation of paxillin, cortactin and VE-cadherin, image analysis was performed on the immunofluorescence channel using imageJ (3). Each area of interest is considered as a particle to analyze using Log3D plugin (Figure S2A, C and E). Bare outline ranges shown in Figure S2B, D and F are the selected areas used for the quantitative analysis. The quantitative analysis was performed on $n = 20$ images at each condition and c-Abl depletion statistically decreases paxillin, cortactin and VE-cadherin intensity in the stimulation conditions of thrombin, S1P and thrombin+S1P ($p < 0.005$). Figure S3, S4, S5 are the images of actin filament (red) and paxillin (green) as shown in Figure 6-8. Paxillin, cortactin and VE-cadherin distribution pattern are taken with Alexa 488 fluorophore. Actin cytoskeleton images are with rhodamine-conjugated phalloidin. Left group is for cells transfected with scrambled siRNA and right for those transfected with c-Abl siRNA.

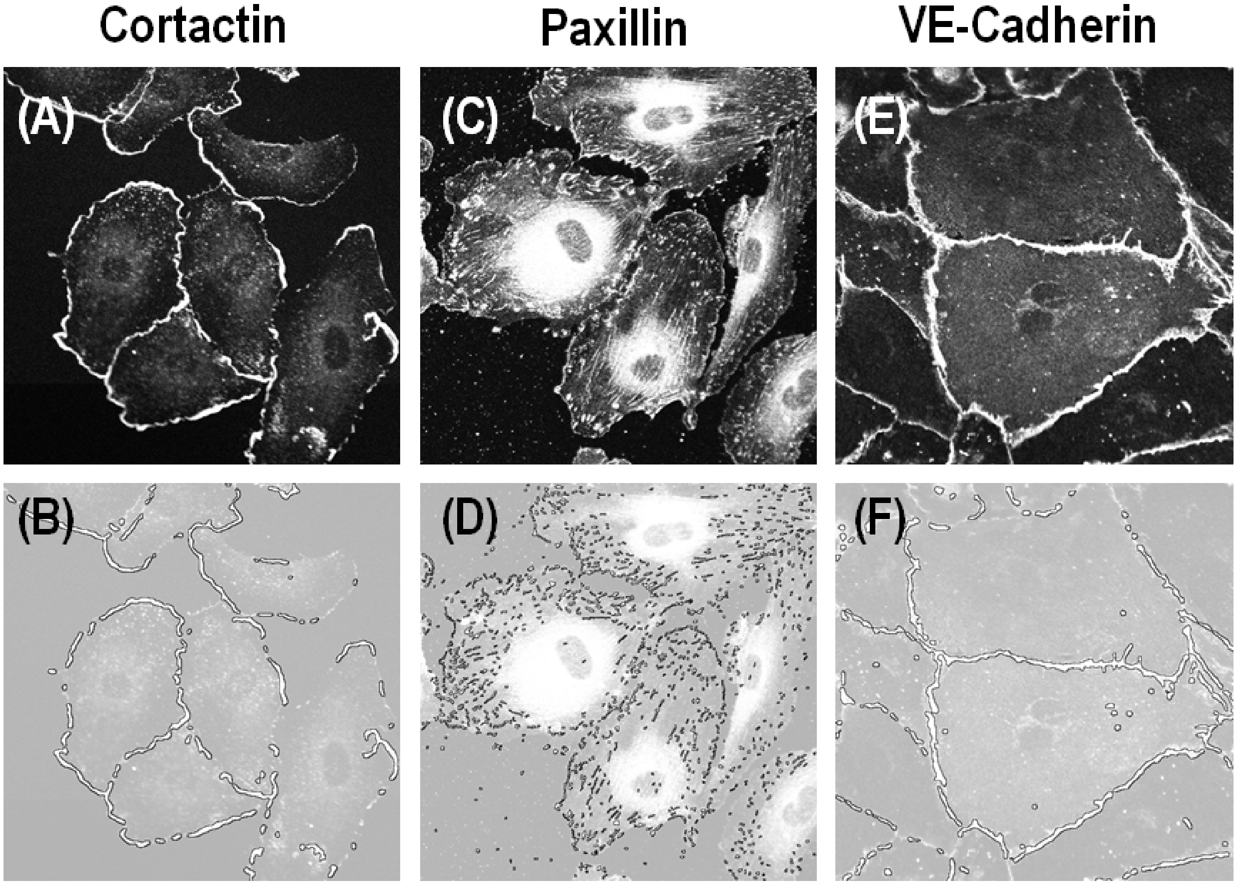

Figure S2. Quantitative analysis on immunofluorescence images of cortactin, paxillin and VE-cadherin (A, C and E) and bare ranges outlined for analyzing proteins of interest (B, D and F)

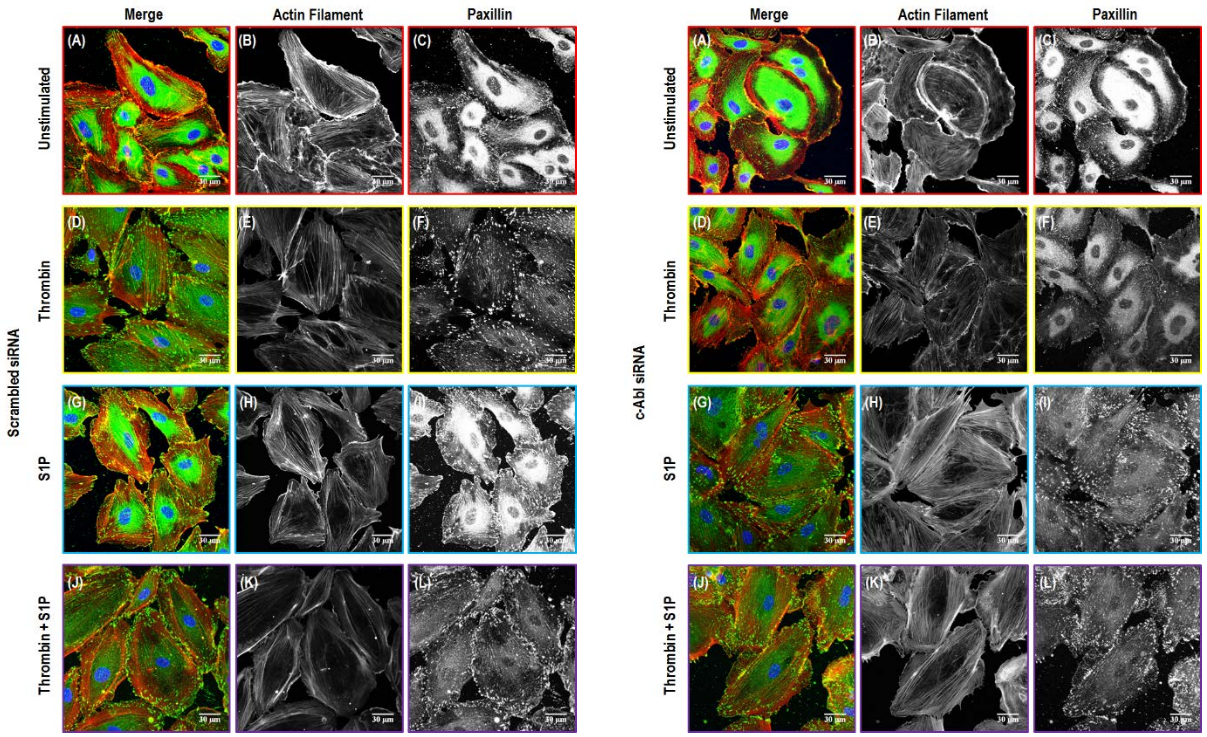

Figure S3. Merged images between actin filament (red) and paxillin (green). Paxillin distribution pattern are taken with Alexa 488 fluorophore and actin cytoskeleton images are with rhodamine-conjugated phalloidin. Left group is for cells transfected with scrambled siRNA and right for those transfected with c-Abl siRNA.

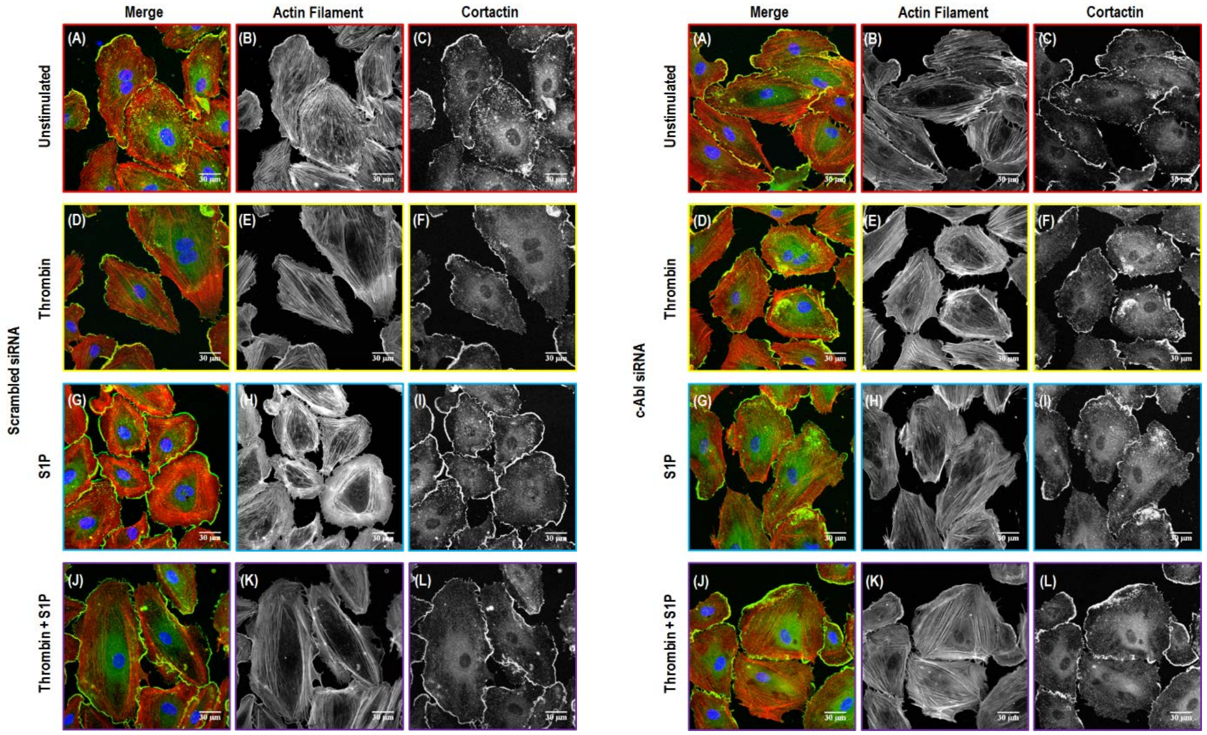

Figure S4. Merged images between actin filament (red) and cortactin (green). Cortactin distribution pattern are taken with Alexa 488 fluorophore and actin cytoskeleton images are with rhodamine-conjugated phalloidin. Left group is for cells transfected with scrambled siRNA and right for those transfected with c-Abl siRNA.

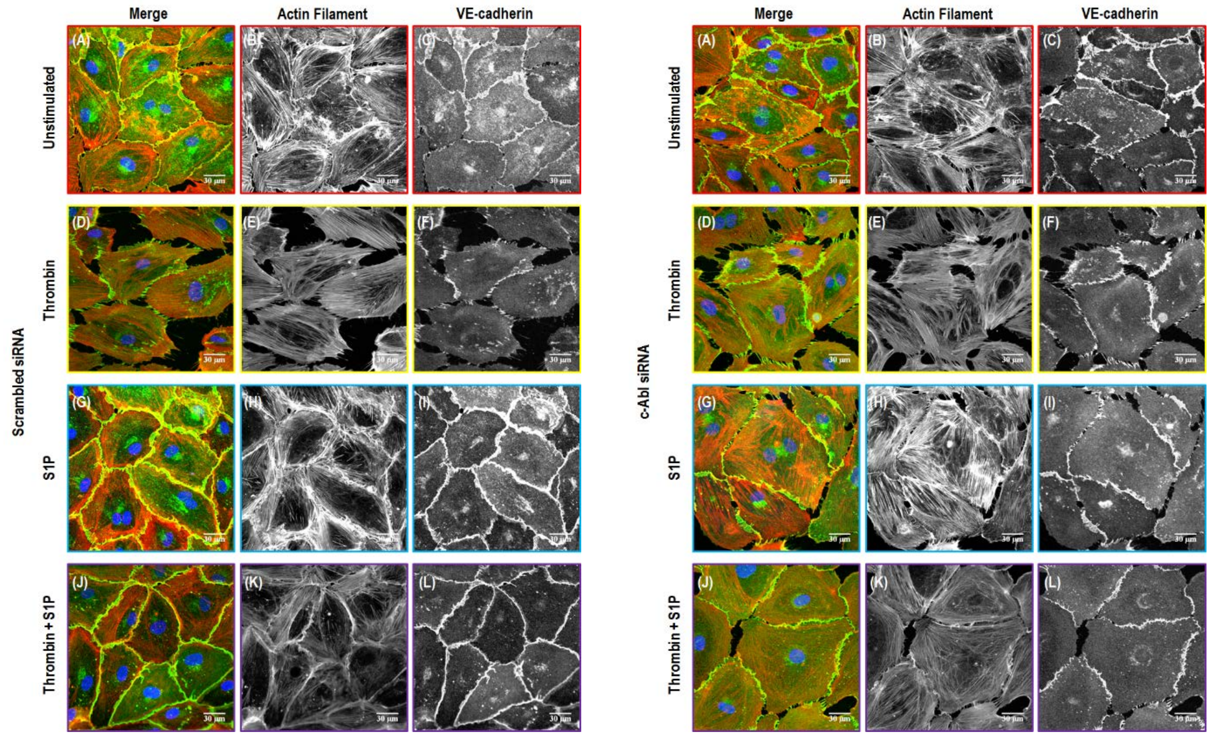

Figure S5. Merged images between actin filament (red) and VE-cadherin (green). VE-cadherin distribution pattern are taken with Alexa 488 fluorophore and actin cytoskeleton images are with rhodamine-conjugated phalloidin. Left group is for cells transfected with scrambled siRNA and right for those transfected with c-Abl siRNA.

References

1. Sneddon, I.N, *The relation between load and penetration in the axisymmetric boussinesq problem for a punch of arbitrary profile*. International Journal of Engineering Science, 1965. **3**(1): p. 47-57.
2. Carl, P. and Schillers, H., *Elasticity measurement of living cells with an atomic force microscope: data acquisition and processing*. Pflugers Archiv-European Journal of Physiology, 2008. **457**(2): p. 551-559.
3. Elosegui-Artola, A., Jorge-Penas, A., Moreno-Arotzena, O., Oregi, A., Lasa, M., Garcia-Aznar, J. M., De Juan-Pardo, E. M., and Aldabe, R., *Image Analysis for the Quantitative Comparison of Stress Fibers and Focal Adhesions*. Plos One, 2014. **9**(9).
